# IFN-λ3 Inhibits HIV Infection of Macrophages through the JAK-STAT Pathway

**DOI:** 10.1371/journal.pone.0035902

**Published:** 2012-04-27

**Authors:** Man-Qing Liu, Dun-Jin Zhou, Xu Wang, Wang Zhou, Li Ye, Jie-Liang Li, Yi-Zhong Wang, Wen-Zhe Ho

**Affiliations:** 1 Division of Virology, Wuhan Center for Disease Prevention & Control, Wuhan, Hubei, People's Republic of China; 2 The Center for Animal Experiment Animal and Biosafety Level III Laboratory, State Key Laboratory of Virology, Wuhan University, Wuhan, Hubei, People's Republic of China; 3 Department of Pathology and Laboratory Medicine, Temple University School of Medicine, Philadelphia, Pennsylvania, United States of America; University of Nebraska Medical Center, United States of America

## Abstract

**Background:**

Interferon lambda 3 (IFN-λ3) is a newly identified cytokine with antiviral activity, and its single nucleotide polymorphisms are strongly associated with the treatment effectiveness and development of chronic hepatitis C virus infection. We thus examined the potential of IFN-λ3 to inhibit HIV replication and the possible mechanisms of the anti-HIV action by IFN-λ3 in human macrophages.

**Principal Findings:**

Under different conditions (before, during, and after HIV infection), IFN-λ3 significantly inhibited viral replication in macrophages, which was associated with the induction of multiple antiviral cellular factors (ISG56, MxA, OAS-1, A3G/F and tetherin) and IFN regulatory factors (IRF-1, 3, 5, 7 and 9). This anti-HIV action of IFN-λ3 could be compromised by the JAK-STAT inhibitor. In addition, IFN-λ3 treatment of macrophages induced the expression of toll-like receptor 3 (TLR3) and two key adaptors (MyD88 and TRIF) in type I IFN pathway activation. However, HIV infection compromised IFN-λ3-mediated induction of the key elements in JAK-STAT signaling pathway.

**Conclusions:**

These data indicate that IFN-λ3 exerts its anti-HIV function by activating JAK-STAT pathway-mediated innate immunity in macrophages. Future *in vivo* studies are necessary in order to explore the potential for developing IFN-λ3-based therapy for HIV disease.

## Introduction

Interferon lambdas (IFN-λs) are a class of newly identified members of IFN family. IFN-λ subfamily is comprised of three structurally related cytokines (IFN-λ1, IFN-λ2, IFN-λ3), which are also called interleukin-29 and interleukin-28A/B (IL-29, IL-28A, IL-28B), respectively [1], [2]. IFN-λ could be activated following viral infections or activation of toll-like receptors (TLRs) [3], [4]. The expression of IFN-λ can induce antiviral factors to suppress the replication of broad spectrum of viruses [4]. IFN-λ functionally resembles type I IFNs, inducing antiviral protection *in vitro* [1], [2], [5], [6] as well as *in vivo* [7]. However, unlike type I IFN receptors that are broadly expressed on most cell types, including cells in the brain, the expression pattern of the IFN-λ receptors is more cell-specific. Thus IFN-λ has fewer side effects than IFN-α [8], which is considered as a significant advantage over IFN-α. IFN-λ binds to its two receptors, which activates JAK-STAT signaling pathway, resulting in the phosphorylation of STAT proteins and forming of ISGF3 complex [9]. The formed ISGF3 complex binds to the IFN-stimulated response element (ISRE) and induces host responses to viral infections [10]. The potential clinical importance of IFN-λ as a novel antiviral therapeutic agent has recently become apparent. Several groups [11]–[14] reported that the endogenous IFN-λ system is associated with treatment-induced clearance of hepatitis C virus (HCV). This finding has further promoted interest in studying the antiviral mechanisms of IFN-λ, particularly IFN-λ3.

Although it has been reported that two members of IFN-λ family, IFN-λ1 and IFN-λ2, could inhibit HIV replication in macrophages [6], it is unclear, however, whether IFN-λ3 possesses the anti-HIV function as well. In this study, we examined whether IFN-λ3 has the antiviral ability against HIV infection of macrophages. We also examined the mechanisms involved in IFN-λ3-mediated actions against HIV.

## Materials and Methods

### Cells and viruses

Peripheral blood samples were obtained from healthy donors and tested for HIV antibody. The Institutional Review Board of the Temple University approved this research, and written informed consents were obtained from all the subjects. All investigations have been conducted according to the principles expressed in the Declaration of Helsinki. PBMCs were isolated by Ficoll gradient, and further incubated with Dulbecco's modified Eagle medium (DMEM) in gelatin-coated flasks for 45 min in 37°C to collect monocytes. After detachment with 10 mM EDTA, monocytes were washed with DMEM and resuspended in 10% DMEM including 10% fetal bovine serum, glutamine (2 mmol/ml), penicillin (100 U/ml), streptomycin (100 µg/ml), and nonessential amino acids. Monocytes purification were determined by nonspecific esterase staining and fluorescence-activated cell sorting analysis with a monoclonal antibody against CD14 (Leu-M3) and low-density lipoprotein specific for monocytes and macrophages [6]. Monocytes were seeded in 96-well-plate (10^5^ cells/well), or 48-well-plate (2.5×10^5^ cells/well), and differentiated to macrophages after cultured for 7 days. HIV Bal strain (R5) was obtained from the AIDS Research and Reference Reagent Program, National Institutes of Health (Bethesda, MD).

### Reagents

Recombinant human IFN-λ1 and IFN-λ2 were purchased from PeproTech Inc. (Rocky Hill, NJ). Recombinant human IFN-λ3 was purchased from R&D Systems, Inc. (Minneapolis, MN), the purity of IFN-λ3 is similar to that of IFN-λ1 and IFN-λ2. JAK inhibitor I was purchased from EMD Chemicals, Inc. (Gibbstown, NJ), and dissolved in 1% dimethyl sufoxide (DMSO). HIV p24 monoclonal antibody was obtained from NIH AIDA Research & Reference Reagent Program.

### HIV infection and IFN-λ treatment

Seven-day-cultured macrophages were incubated with IFN-λ either 24 h prior to HIV Bal infection, or simultaneously with HIV infection, or 4 days after HIV infection. The productive HIV infection was determined at day 12 postinfection. In the experiments where the dose and time effects of IFN-λ3 were examined, IFN-λ3 was added to the cultures 4 days postinfection and continuously presented in the cultures. In order to determine the role of JAK-STAT pathway in the IFN-λ3 action against HIV, JAK inhibitor I was added to cell culture 1 h prior to IFN-λ3 treatment.

### Reverse transcriptase (RT) activity assay

HIV RT activity was determined with the technique of Willey et al. [15] with some modifications [6], [16]. Briefly, 10 µl of supernatant collected from HIV-infected macrophages cultures were added to 50 µl of cocktail containing poly (A), oligo (dT), MgCl_2_, Nonidet P-40, and [^32^P] dTTP and incubated overnight at 37°C. Thirty microliters of the reaction mixture was spotted on DE 81 paper and air dried. The filters were then washed for four times in fresh standard saline citrate (SSC, contained with 0.3 mol/liter NaCl, 0.03 mol/liter sodium citrate, PH 7) and 100% ethanol. After dried, the filters were placed into the scintillation vials and measured for radioactivity by liquid scintillation analyzer (PerkinElmer Tri-Carb 2810TR, Downers Grove, IL60515, USA).

### RNA extraction and real-time RT-PCR

Total cellular nucleotide was extracted from macrophages using TRI® Reagent (SIGMA-ALDRICH, Inc. St. Louis, MO 63178, USA) as described previously [6]. Total RNA (1 µg) was subjected to the reverse transcription using the RT system (Promega, Madison, WI) for 1 h at 42°C. The resulting cDNA was used as a template for real-time PCR quantification. Using the iQ SYBR Green supermix (Bio-Rad Laboratories, Hercules, California), messenger RNAs (mRNAs) in macrophages were detected for antiviral factors (interferon-stimulated gene 56 (ISG-56), myxovirus resistance-1 (MxA), obstructive sleep apnoea-1 (OAS-1), APOBEC3G/APOBEC3F (A3G/F), tetherin, IFN regulatory factor (IRF)-1, IRF-3, IRF-5, IRF-7, IRF-9, toll-like receptor (TLR)-3, TLR-7, TLR-9, IFN-λ3 receptors (IL-28Rα, IL-10Rβ), IFN-λ3 signal pathway cytokines (Myeloid differentiation primary response gene 88 (MyD88), TIR-domain-containing adapter-inducing interferon-β (TRIF), signal transducer and activator of transcription (STAT-1/2)) and glyceraldehydes 3-phosphate dehydrogenase (GAPDH). The primers used for the parameters above were listed in Table 1. The levels of GAPDH mRNA were used as an endogenous reference to normalize the quantities of target mRNA and presented as the change in induction relative to that of untreated control cells.

**Table 1:** Primers for Real-time PCR.

Primer	Sense	Antisense
GAPDH	5′-GGTGGTCTCCTCTGACTTCAACA-3′	5′-GTTGCTGTAGCCAAATTCGTTGT-3′
TLR-3	5′-AGCCACCTGAAGTTGACTCAGG-3′	5′-CAGTCAAATTCGTGCAGAAGGC-3′
TLR-7	5′-AAAATGGTGTTTCCAATGTGG-3′	5′-GGCAGAGTTTTAGGAAACCATC-3′
TLR-9	5′-TACCAACATCCTGATGCTAGACTC-3′	5′-TAGGACAACAGCAGATACTCCAGG-3′
IRF-1	5′-TGAAGCTACAACAGATGAGG-3′	5′-AGTAGGTACCCCTTCCCATC-3′
IRF-3	5′-ACCAGCCGTGGACCAAGAG-3′	5′-TACCAAGGCCCTGAGGCAC-3′
IRF-5	5′-AAGCCGATCCGGCCAA-3′	5′-GGAAGTCCCGGCTCTTGTTAA-3′
IRF-7	5′-TGGTCCTGGTGAAGCTGGAA-3′	5′-GATGTCGTCATAGAGGCTGTTGG-3′
IRF-9	5′-GCATCAGGCAGGGCACGCTGCACC-3′	5′-GCCTGCATGTTTCCAGGGAATCCG-3′
MyD88	5′-GCACATGGGCACATACAGAC-3′	5′-TGGGTCCTTTCCAGAGTTTG-3′
TRIF	5′-CTCACCTGACCCCCTCCT-3′	5′-AATTTCTGTTCCGATGATGATTC-3′
STAT-1	5′-GTGGAAAGACAGCCCTGCAT-3′	5′-ACTGGACCCCTGTCTTCAAGAC-3′
STAT-2	5′-CCCCATCGACCCCTCATC-3′	5′-GAGTCTCACCAGCAGCCTTGT-3′
IL-28Rα	5′-ACCTATTTTGTGGCCTATCAGAGCT-3′	5′-CGGCTCCACTTCAAAAAGGTAAT-3′
IL-10Rβ	5′-GGCTGAATTTGCAGATGAGCA-3′	5′-GAAGACCGAGGCCATGAGG-3′
ISG-56	5′-TTCGGAGAAAGGCATTAGA-3′	5′-TCCAGGGCTTCATTCATAT-3′
MxA	5′-GCCGGCTGTGGATATGCTA-3′	5′-TTTATCGAAACATCTGTGAAAGCAA-3′
OAS-1	5′-AGAAGGCAGCTCACGAAACC-3′	5′-CCACCACCCAAGTTTCCTGTA-3′
A3G	5′-TCAGAGGACGGCATGAGACTTAC-3′	5′-AGCAGGACCCAGGTGTCATTG-3′
A3F	5′-TTCGAGGCCAGGTGTATTCC-3′	5′-GGCAGCTGGTTGCCACAGA-3′
Tetherin	5′-AAGAAAGTGGAGGAGCTTGAGG-3′	5′-CCTGGTTTTCTCTTCTCAGTCG-3′

### Indirect immunofluorescence assay for HIV p24

HIV Bal-infected macrophages treated with or without IFN-λ3 were washed with phosphate-buffered saline (PBS) and fixed in 4% paraformaldehyde (EM Science) for 20 min at room temperature. The cells were then permeabilized with 0.2% Triton for 10 min, and blocked in blocking solution (10% fetal calf serum, 1% penicillin/streptomylin, and 0.05% sodium azide in MEM) for 1 h. HIV p24 antigen was detected using mouse monoclonal anti-HIV-p24 antibody and goat-anti mouse antibody conjugated with Alexa Fluor 488 (Invitrogen). The cell nuclei were counterstained with Hoechst dye. Microscopic analysis was performed with a fluorescence microscope (Olympus).

### Statistical analysis

Where appropriate, data were expressed as means ± standard deviations (SD) of at least three samples replicates. Data analysis was performed using a 2-tailed Student's t-test. Statistical analyses were performed with Graphpad Instat Statistical Software (Graphpad Software Inc., San Diego, CA), and the statistical significance was defined as *P<0.05*.

## Results

### IFN-λ3 inhibits HIV replication in macrophages

**Figure 1:**
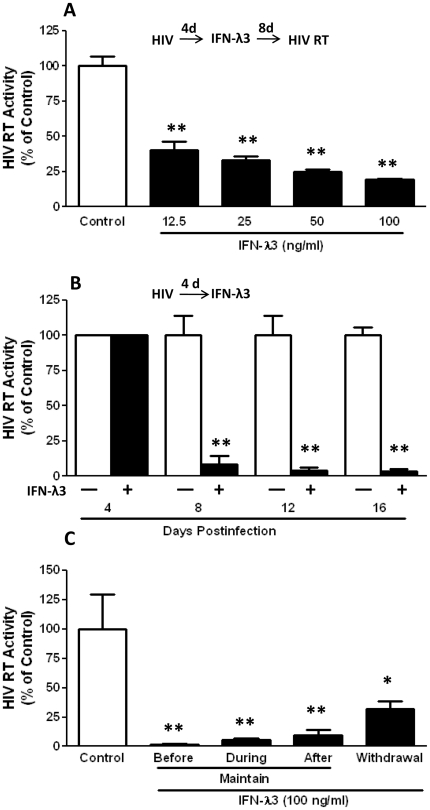
Effect of IFN-λ3 on HIV infection of human macrophages. **A.** Dose-dependent effect of IFN-λ3 on HIV infection. Seven-day-cultured macrophages were infected with HIV Bal strain for 4 days, and then treated with IFN-λ3 at indicated concentrations. HIV RT activity in supernatant was analyzed at day 12 postinfection. **B.** Time-dependent effect of IFN-λ3 on HIV infection. Seven-day-cultured macrophages were infected with HIV Bal strain for 4 days and then treated with IFN-λ3 at concentration of 100 ng/ml. HIV RT activity in the supernatant at indicated time point was examined. **C.** Effect of IFN-λ3 on HIV infection and replication under four different conditions. Macrophages were pre-treated with IFN-λ3 (100 ng/ml) for 24 h, and then infected with HIV Bal strain (Before), or co-incubated with IFN-λ3 (100 ng/ml) and HIV Bal strain at the same time (During), or 4 days after HIV infection, macrophages under the conditions above were treated with IFN-λ3 (100 ng/ml) with or without IFN-λ3 withdrawal. The levels of HIV RT activity in supernatant were analyzed at day 12 postinfection. The data shown were the mean ± standard deviation of triplicate culture, and expressed as % of control (without IFN-λ3 treatment, the mean value of which is defined as 100) (*P<0.05, **P<0.01).

**Figure 2:**
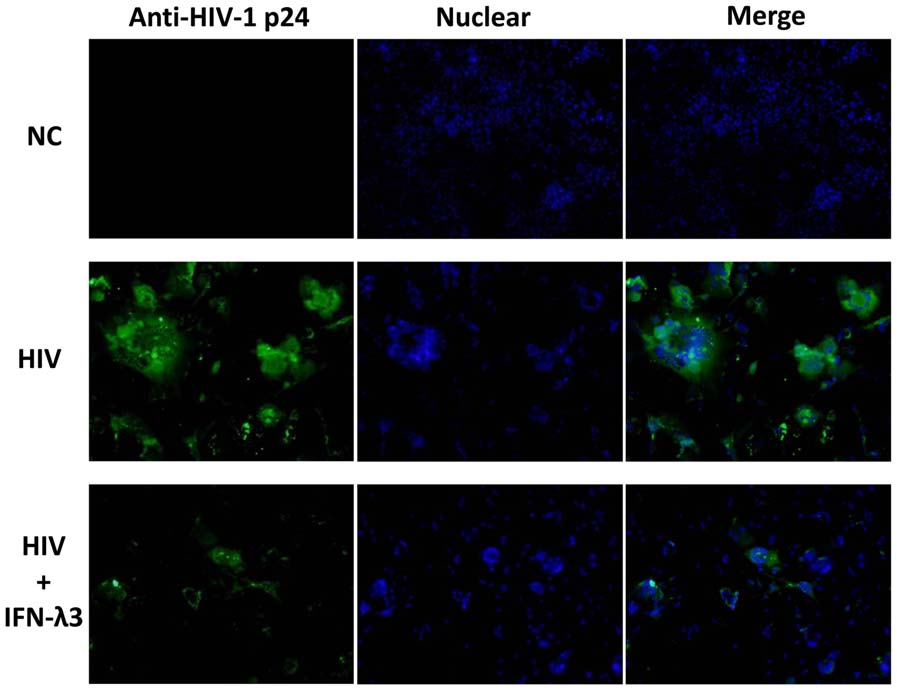
Indirect immunofluorescence assay for HIV p24. Seven-day-cultured macrophages were infected with or without HIV Bal strain for 2 h and cultured for 12 days in the presence or absence of IFN-λ3 (100 ng/ml). Cells were then washed and fixed in 4% paraformaldehyde fixative and treated with 0.2% triton. HIV p24 in infected macrophages was stained by HIV p24 antiserum (green color) and cell nuclei was stained by Hoechst and observed through fluorescence microscope. (NC, Negative Control; PC, Positive Control).

We first examined the effect of IFN-λ3 on HIV replication in macrophages. When added to HIV-infected macrophage cultures, IFN-λ3 significantly inhibited HIV RT activity in a dose (Fig. 1A) and time (Fig. 1B) dependant fashion. This inhibitory effect of IFN-λ3 on HIV was observed under three different treatment conditions (Fig. 1C). The highest inhibition of HIV was observed in macrophage cultures pretreated with IFN-λ3 (prior to HIV infection) (Fig. 1C). To maintain IFN-λ3 in HIV-infected cell cultures was necessary, as the withdrawal of IFN-λ3 from infected cultures resulted in a viral rebound (Fig. 1C). HIV inhibition by IFN-λ3 was also confirmed by indirect immunofluorescence staining with antibody against HIV p24 antigen (Fig. 2).

### IFN-λ3 induces key elements in JAK-STAT pathway

**Figure 3:**
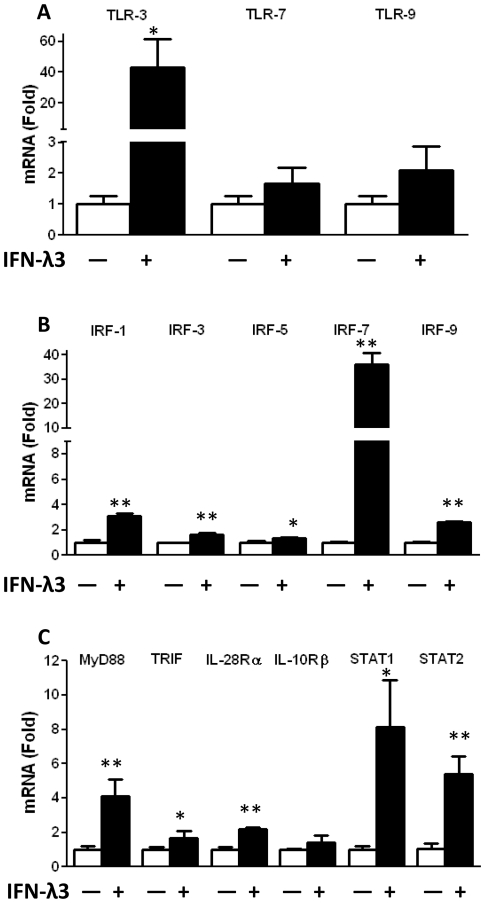
Effect of IFN-λ3 on the key elements in JAK-STAT pathway in uninfected macrophages. Seven-day-cultured macrophages were treated with or without IFN-λ3 (100 ng/ml) for 24 h. Total nucleotide were extracted from macrophages and subjected to the real-time reverse transcription polymerase chain reaction (RT-PCR) assay for mRNA expression of TLRs (**A**), IRFs (**B**) and the key elements in IFN-λ3 signaling pathway (**C**). The data shown were the mean ± standard deviation of triplicate culture (*P<0.05, **P<0.01).

**Figure 4:**
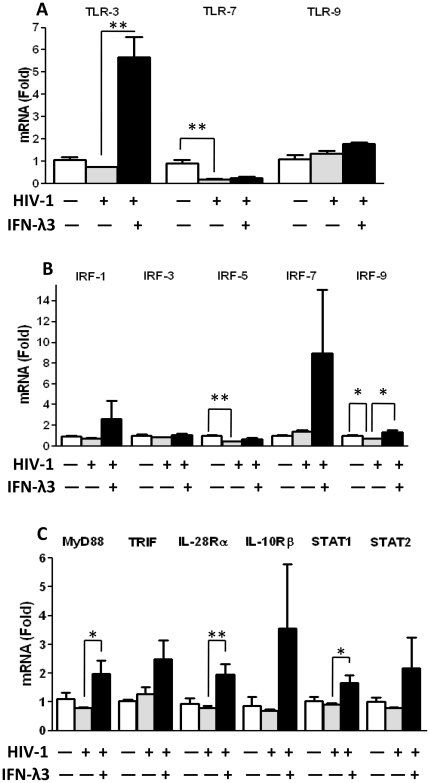
Effect of IFN-λ3 on the key elements in JAK-STAT pathway in HIV infected macrophages. Seven-day-cultured macrophages were infected with HIV Bal strain for 2 h. IFN-λ3 (100 ng/ml) was added to the cultures 4 days postinfection for 24 h. Total nucleotide were extracted from macrophages and subjected to the real-time reverse transcription polymerase chain reaction (RT-PCR) assay for mRNA expression of TLRs (**A**), IRFs (**B**) and the key elements in IFN-λ3 signaling pathway (**C**). Seven-day-cultured macrophages neither infected with HIV Bal strain nor treated with IFN-λ3 were set as control for a fair comparison with that in Figure 3. The data shown were the mean ± standard deviation of triplicate culture (*P<0.05, **P<0.01).

To determine the mechanism of IFN-λ3-mediated anti-HIV activity in macrophages, we examined the effect of IFN-λ3 on the expression of TLRs as well as several key elements in IFN-λ signaling pathway in both uninfected and infected macrophages. We first determined whether IFN-λ3 treatment modulates TLR (TLR3, 7, 9) expression in macrophages. IFN-λ3 treatment of uninfected macrophages could induce the expression of TLR7 and TLR9 (Fig. 3A). More significantly, IFN-λ3 induced TLR3 expression in uninfected macrophages as much as 40 folds (Fig. 3A). We next examined whether IFN-λ3 upregulates the expression of IFN regulatory factors (IRFs). As shown in Fig. 3B, IFN-λ3 induced the expression of several key IRFs, particularly IRF7 in uninfected macrophages (Fig. 3B). Because IFN-λ, through binding to IL-28Rα and IL-10Rβ, activates JAK-STAT pathway [17], and TLR activation induces type I IFN expressions [18], we further examined the impact of IFN-λ3 on type I IFN signaling pathway. As shown in Fig. 3C, the expression of IL-28Rα and STAT-1/2 was significantly induced by IFN-λ3 in uninfected macrophages. In addition, IFN-λ3 treatment of uninfected macrophages enhanced the expression of two key adaptors (MyD88 and TRIF) in type I IFN pathway activation (Fig. 3C). Because HIV infection/replication could have a negative effect on the expression of the antiviral cellular factors, we examined the impact of HIV on IFN-λ3-mediated induction of the key elements in JAK-STAT pathway in infected macrophages. As shown in Fig. 4, although IFN-λ could induce the expression of TLRs as well as other antiviral factors in infected macrophages, the degree of the induction was lower (Fig. 4) than that in uninfected cells (Fig. 3).

### IFN-λ3 is more potent in suppressing HIV than IFN-λ1 and IFN-λ2

**Figure 5:**
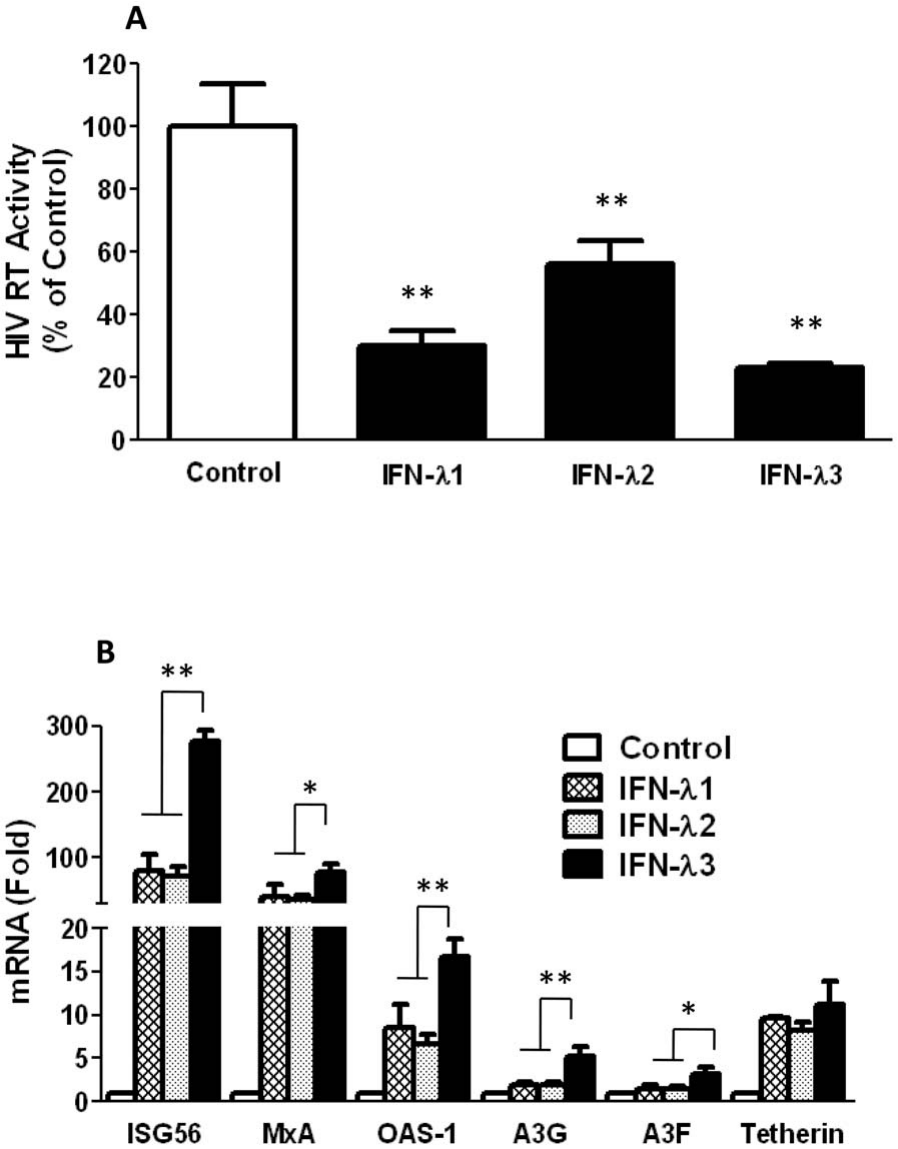
Effects of IFN-λ1, IFN-λ2 and IFN-λ3 on HIV. **A.** Seven-day-cultured macrophages were infected with HIV Bal strain for 4 days and then treated with or without IFN-λ1/2 (100 ng/ml) or IFN-λ3 (100 ng/ml) for 8 days. HIV RT activity in the supernatant was analyzed at day 12 postinfection. The data were expressed as HIV RT levels relative (%) to control (without IFN-λ treatment, which defined as 100). **B.** Seven-day-cultured macrophages were treated with or without IFN-λ1/2 (100 ng/ml) or IFN-λ3 (100 ng/ml) for 24 h, and total nucleotide of macrophages was extracted for real-time RT-PCR analysis of the mRNA expression of ISG-56, MxA, OAS-1, A3G/F, tetherin and GAPDH. The data were expressed as mRNA levels for the anti-HIV factors relative (folds) to the control (without IFN-λ treatment, which is defined as 1). (*P<0.05, **P<0.01).

IFN-λ1 or IFN-λ2 has been shown to inhibit HIV replication in macrophages [6], we thus compared the anti-HIV ability of IFN-λ3 with IFN-λ1 or IFN-λ2. As shown in Fig. 5A, the inhibitory effect of IFN-λ3 on HIV in macrophages is more potent than that of IFN-λ1 or IFN-λ2. In addition, IFN-λ3 was the most potent inducer of cellular antiviral factors (ISG-56, MxA, OAS-1, A3G/F and tetherin) among the three IFN-λ family members (Fig. 5B).

### The anti-HIV activity of IFN-λ3 is dependent on JAK-STAT pathway

**Figure 6:**
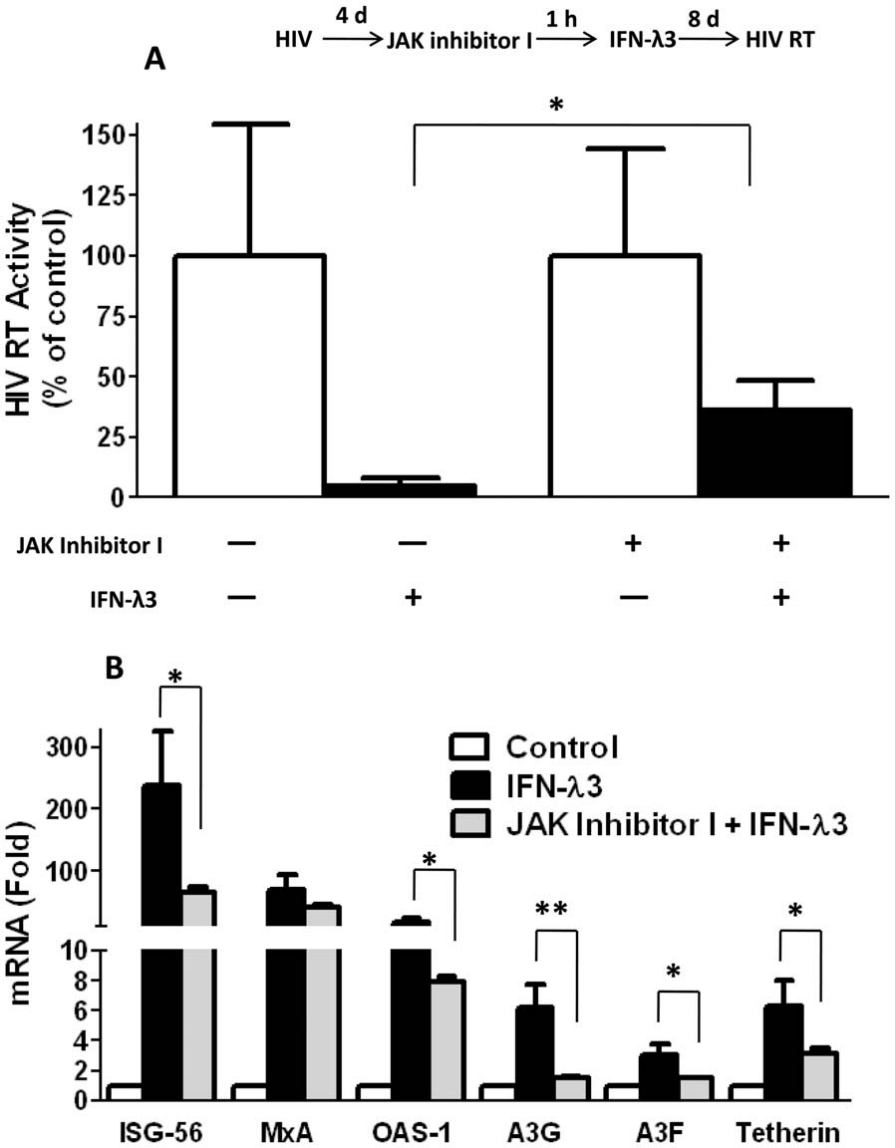
Effect of JAK-STAT inhibitor on the IFN-λ3 actions. **A.** Effect of JAK-STAT inhibitor on the anti-HIV action of IFN-λ3. Macrophages were infected with HIV Bal strain for 4 days, and then incubated with JAK inhibitor I (1 µM) for 1 h prior to IFN-λ3 (100 ng/ml) treatment. HIV RT activity in supernatant was measured at day 12 postinfection. **B.** Effect of JAK-STAT inhibitor on IFN-λ3-mediated induction of cellular antiviral factors. Macrophages were incubated with JAK inhibitor (1 µM) for 1 h prior to IFN-λ3 (100 ng/ml) treatment for 24 h. The expression of ISG-56, MxA, OAS-1, A3G/F and tetherin was indicated as the increase in induction (folds) relative to without IFN-λ3 treated cells, which is normalized to GAPDH levels. Values were expressed as mean ± standard deviation of triplicate culture (* P<0.05, ** P<0.01).

In order to explore the mechanisms of the anti-HIV activity of IFN-λ3, we examined the role of JAK-STAT pathway in the IFN-λ3 action against HIV. As shown in Fig. 6A, the anti-HIV activity of IFN-λ3 was found to be significantly suppressed by the inhibitor (JAK inhibitor I) of JAK-STAT pathway. In addition, JAK inhibitor I treatment compromised the inducing effects of IFN-λ3 on several key antiviral cellular factors, including ISG-56, MxA, OAS-1, A3G/F and tetherin (Fig. 6B).

## Discussion

Compared to IFN-λ1 or IFN-λ2, IFN-λ3 is thought to be more important for anti-HCV therapy, as its genetic variation of three single-nucleotide polymorphisms (SNPs) (rs12979860, rs12980275, and rs8099917) in upstream of the IFN-λ3 (IL-28B) gene is highly associated with treatment outcomes of Peg-IFNα-2a plus ribavirin therapy [11]–[14]. Although the anti-HIV function of IFN-λ1 or IFN-λ2 has been demonstrated [6], it remains to be determined whether IFN-λ3 has the ability to suppress HIV. In the present study, we have provided the experimental evidence that similar to IFN-λ1 and IFN-λ2, IFN-λ3 treatment resulted in the inhibition of HIV infection of macrophages. When macrophages were pretreated once with IFN-λ3, a nearly complete inhibition of HIV of macrophages was observed (Fig. 1). The presence of IFN-λ3 in the cultures is necessary to maintain HIV suppression, as the withdrawal of IFN-λ3 from the cultures resulted in a rebound of viral replication (Fig. 1C). This inhibitory effect of IFN-λ3 treatment was seen even after HIV infection had been initiated in macrophages (Fig. 1). We also demonstrated that IFN-λ3 appeared to be more potent in inhibiting HIV replication in macrophages than IFN-λ1 or IFN-λ2 (Fig. 5). This enhanced ability to inhibit HIV could be due to its stronger effects on several key cellular antiviral factors in macrophages than IFN-λ1 or IFN-λ2 (Fig. 5B).

IFN-λ functionally resembles type I IFNs in antiviral protection [19]. Despite of the overlapping antiviral functions of IFN-λ with type I IFNs, the more restriction pattern of IFN-λ receptor (IL-28Rα) expression suggests that IFN-λ has a more specialized role in host antiviral defense against viruses in a subset of cells [20], [21]. In addition, the mechanisms by which IFN-λ establishes an antiviral state are not as well characterized as those for IFN-α/β. Our studies showed that IFN-λ3 through multiple mechanisms suppressed HIV infection and replication. We first investigated whether IFN-λ3 activates JAK-STAT signaling pathway, inducing the antiviral state in macrophages. IFN-λ3 treatment of macrophages upregulated the expression of either key factors of JAK-STAT pathway, such as STAT1 and STAT2 (Fig. 3C, 4C), or the known ISGs in the downstream of JAK-SATA pathway, including ISG-56, MxA, and OAS-1 (Fig. 5B). The role of JAK-STAT pathway in the anti-HIV activity of IFN-λ3 was further confirmed by the observation that to block of JAK-STAT signaling pathway by the JAK inhibitor could compromise the IFN-λ3 action on HIV and significantly decrease the expression of IFN-λ3-induced antiviral factors (Fig. 6). We also demonstrated that APOBEC3G and APOBEC3F, the specific anti-HIV cellular factors, were induced by IFN-λ through JAK-STAT pathway (Figs. 5B and 6B). In addition, tetherin, a transmembrane protein that specifically inhibits HIV infection by preventing its release from infected cells, was also induced by IFN-λ through JAK-STAT pathway (Figs. 5B and 6B). Thus, JAK-STAT pathway plays a crucial role in the anti-HIV activity of IFN-λ3 in macrophages, which is in agreement with the previous findings, showing that IFN-λ3, through the activation of JAK-STAT pathway, exerts its anti-HCV function in human hepatocytes [10].

TLRs are crucial in the innate immune responses to pathogens, because they recognize and respond to PAMPs, which leads to activation of intracellular signaling pathways. Among the eleven identified human TLRs, three TLRs (TLR- 3, 7, and 9) play a key role in virus-mediated innate immunity, as they specifically recognizes viral RNA or DNA and initiate antiviral signaling pathways in macrophages [22], [23]. Our studies demonstrated that IFN-λ3 could significantly induce the expression of TLR3 as well as TLR3-associated adaptors such as MyD88 and TRIF (Figs. 3A, 3C, 4A and 4C). These observations provide an additional mechanism for IFN-λ3-mediated anti-HIV action, as TLR3 signaling has a crucial role in early innate immune response to viral infections, including HIV [24].

Our further investigation showed that IFN-λ3 induced the expression of IRFs (IRF-1, 3, 5, 7 and 9) in macrophages infected with or without HIV (Figs. 3B and 4B). The IRFs are important regulators in the type I and III IFN-mediated antiviral immunity. These regulators not only recognize the elements of DNA promoter to modulate type I and III IFN genes, but also regulate the IFN-stimulated response element (ISRE) in some of ISGs, leading to induction of an antiviral state [25], [26]. Recent studies [27]–[29] have further demonstrated an essential role of IRF-3, IRF-5, and IRF-7 in the activation of type I and III IFN expression, which is critical for the production of down-stream antiviral factors such as ISG-56, MxA, OAS-1, tetherin (Fig. 6).

Taken together, our data have provided the compelling evidence that IFN-λ3 through the activation of TLR3 and JAK-STAT pathways inhibited HIV infection of macrophage. Although the precise cellular and molecular mechanisms by which IFN-λ3 inhibits HIV replication remain to be determined, the induction of multiple cellular restriction factors against HIV should account for much of IFN-λ3-mediated anti-HIV activity. These anti-HIV mechanisms of the IFN-λ3 action offer an attractive alternative for HIV treatment, as it would be extremely difficult for HIV to develop resistance to the IFN-λ3 actions that suppress virus at various steps of its replication in the context of host cell innate immunity. However, future studies are needed in order to determine the impact of IFN-λ3 on HIV in *ex vivo* and *in vivo* systems. These additional studies shall explore the potential for developing IFN-λ3-based therapy for people infected with HIV.
